# Keratinocyte growth factor induces matrix metalloproteinase-9 expression and correlates with venous invasion in pancreatic cancer

**DOI:** 10.3892/ijo.2011.1280

**Published:** 2011-12-06

**Authors:** KAZUMITSU CHO, YOKO MATSUDA, JUNJI UEDA, EIJI UCHIDA, ZENYA NAITO, TOSHIYUKI ISHIWATA

**Affiliations:** 1 Departments of Pathology and Integrative Oncological Pathology, Nippon Medical School, 1-1-5 Sendagi, Bunkyo-ku, Tokyo 113-8602; 2 Surgery for Organ and Biological Regulation, Graduate School of Medicine, Nippon Medical School, 1-1-5 Sendagi, Bunkyo-ku, Tokyo 113-8603, Japan

**Keywords:** keratinocyte growth factor, KGF receptor, venous invasion, pancreatic cancer, matrix metalloproteinase-9

## Abstract

Keratinocyte growth factor (KGF), also known as fibroblast growth factor-7, and KGF receptor (KGFR) play important roles in the growth of epithelial cells and are overexpressed in a variety of malignant epithelial tumors, including pancreatic ductal adenocarcinoma (PDAC). We previously reported that co-expression of KGF and KGFR in PDAC is associated with venous invasion, enhanced vascular endothelial growth factor A expression and poor prognosis. Matrix metalloproteinase-9 (MMP-9) is known to participate in the degradation of type IV collagen, which is a primary component of extracellular matrices in the vascular basement membrane. In the present study, we examined the expression and roles of KGF, KGFR and MMP-9 in human PDAC cell lines and tissues. Quantitative real-time polymerase chain reaction analysis demonstrated the expression of MMP-9 mRNA in all eight PDAC cell lines. KGF, KGFR and MMP-9 were, respectively, expressed in 27 (43%), 23 (37%) and 35 (56%) of 63 patients. Each expression of KGF, KGFR or MMP-9 correlated positively with venous invasion. Furthermore, expression of KGF or MMP-9 correlated positively with liver metastasis. KGF-positive cases exhibited shorter survival than KGF-negative cases, while KGFR and MMP-9 expression were unrelated to prognosis. Administration of recombinant human KGF increased MMP-9 expression in PDAC cells, while transient transfection with short hairpin RNAs targeting KGF transcripts reduced MMP-9 expression in PDAC cells. Moreover, recombinant human KGF significantly enhanced migration and invasion of PDAC cells. These findings suggest that KGF and KGFR promote venous invasion via MMP-9 in PDAC, and closely correlate with liver metastasis. The KGF/KGFR pathway may be a critical therapeutic target for PDAC metastasis.

## Introduction

Pancreatic ductal adenocarcinoma (PDAC) is one of the deadliest of all solid malignant tumors and the fourth leading cause of cancer death in the Western world, with an overall 5-year survival rate of only 6% (1). One reason for this poor prognosis is the propensity of PDAC cells to invade adjacent tissues and to metastasize, even during the early stage (2). PDAC often harbors multiple molecular alterations in cancer cells, including activating *KRAS* mutations and loss-of-function mutations in the P16/CDKN2A, TP53, and SMAD4/DPC4 genes (2). Mutations in these four genes are recognized as ‘driver mutations’ in PDAC because they drive neoplastic transformation and tumor progression (3). A high percentage of PDACs also overexpress a number of growth factors and their receptors, including epidermal growth factor (EGF), EGF receptor (EGFR), human epidermal growth factor (HER)-2/c-erbB2, transforming growth factor (TGF)-α, CRIPTO, TGF-β1, vascular endothelial growth factor (VEGF), basic fibroblast growth factor (bFGF/FGF-2), acidic FGF (aFGF/FGF-1), FGF-5, FGF-7 [also known as keratinocyte growth factor (KGF)], and KGF receptor (KGFR)/FGFR2IIIb (4–11). The multiple stepwise alterations in oncogenes and tumor suppressor genes, in conjunction with the overexpression of mitogenic growth factors and their receptors, may contribute to the formation of precancerous lesions in the pancreas (PanIN) and the biological aggressiveness of PDAC (2,12,13).

KGF is a member of the FGF group of heparin-binding polypeptides, which was initially identified in human embryonic lung fibroblasts (14,15). KGF is synthesized by mesenchymal cells and T lymphocytes, and acts predominantly on epithelial cells in a paracrine manner (16). KGF is expressed in a variety of tissues, including the lung, prostate, mammary gland, digestive tract, bladder, and skin, and has been implicated in organ development and homeostasis (16). KGF also stimulates the growth of the gastrointestinal tract mucosa, and KGF-expressing transgenes exhibit pancreatic ductal hyperplasia (17,18). KGF mRNA levels have been substantially higher in pancreatic and colorectal cancer specimens than in the corresponding normal tissues (11,19).

KGF binds to a specific cell-surface receptor, KGFR, also known as the FGFR2 IIIb isoform (20,21). KGFR and FGFR2IIIc are splicing variants of the FGFR2 gene, and they differ from each other in the carboxy-terminal half of the third immunoglobulin-like region of the extracellular domain (22). KGFR is localized in epithelial cells, while FGFR2 IIIc is mainly localized in mesenchymal cells. KGFR and FGFR2 IIIc exhibit different ligand-binding specificities. FGF-1, -3, -7, -10, and -22 reportedly bind to FGFR2 IIIb with high affinity, while FGF-1, -2, -4, -6, -9, -17, and -18 bind to FGFR2 IIIc with high affinity (20,21). KGFR mRNA is expressed in many organs, including breast, colon, stomach and esophagus, pancreas, prostate, oral mucosa, and uterus (23,24). Loss of FGFR2 IIIb expression has been associated with the activation of FGFR2 IIIc expression, and/or a shift to more virulent behavior (25).

Degradation of basement membranes and extracellular matrices is an essential process in the invasion and metastasis of malignant tumors. Matrix metalloproteinases (MMPs), are potent proteolytic enzymes that play key roles in this process (26). One of the first steps of cancer invasion is basement membrane degradation. MMP-2 (gelatinase A) and MMP-9 (gelatinase B) play important roles in destroying the basement membrane of tumor vessels, and their expression correlates with metastasis (27). MMP-9 cleaves type IV collagen and gelatin, which are the principal structural components of the vascular basement membrane. Expression of MMP-9 has been reported in breast, colon, and lung cancers, as well as skin tumors, and the expression of both MMP-2 and MMP-9 has been correlated with local invasion of the tumor, lymph node metastasis, and survival rates (28). In PDAC, MMP-9 expression is correlated with lymph node involvement and distant metastasis (29). Furthermore, MMP-9 expression is reportedly correlated with (or tends to correlate with) shorter overall survival in PDAC patients (30,31).

We previously reported that the co-expression of KGF and KGFR in PDAC is associated with venous invasion and poor prognosis (32). Although a previous report has shown that VEGF-A expression is closely involved in the KGF/KGFR pathway in PDAC, there have been no reports about the relation between KGF/KGFR and MMPs (32). In this study, we investigated the expression and roles of KGF/KGFR and MMP-9 in human PDAC cell lines and tissues. We now report that the expression of KGF/KGFR and MMP-9 is correlated with venous invasion, and that MMP-9 expression is regulated by KGF/KGFR. The KGF/KGFR pathway might be a potent therapeutic target for PDAC metastasis.

## Materials and methods

### Materials

The following were purchased: Isogen from Nippon Gene (Tokyo, Japan); a Takara RNA PCR kit (AMV) Ver. 3.0 and pBAsi-hU6 Neo DNA vector from Takara Biotech. (Tokyo, Japan); RNeasy mini kit from Qiagen GmbH (Hilden, Germany); Transcriptor First Strand cDNA Synthesis kit and LightCycler FastStart DNA Master SYBR Green I, FuGENE HD transfection reagent from Roche Diagnostics GmbH (Mannheim, Germany); goat polyclonal anti-KGF antibodies and recombinant human KGF (rhKGF) from R&D Systems Inc. (Westerville, OH); a Histofine Simple Stain Max PO (G), (R), or (M) kit from Nichirei Biosciences, Inc. (Tokyo, Japan); mouse monoclonal anti-MMP-9 antibodies from Daiichi Fine Chemical Co., Ltd. (Toyama, Japan); Human Tissue Microarray 1 and Human Digestive Tissue Sets from Novagen (Darmstadt, Germany); Silane-coated slides and malinol mounting medium from Muto Pure Chemicals Co., Ltd. (Tokyo, Japan); Transwell permeable supports from Life Sciences (Acton, MA); Matrigel from BD Biosciences (Franklin Lakes, NJ). All other chemicals and reagents were purchased from Sigma Chemical Corp. (St. Louis, MO).

### Patients and tissues

Tissues from 63 patients with invasive PDAC were obtained for this study. These patients received treatment at Nippon Medical School Hospital (Tokyo, Japan) from 1995 to 2004. None of the patients received preoperative chemotherapy or radiotherapy. The patients consisted of 40 males and 23 females, whose median age was 63, range, 35–84 years. The clinicopathologic stage was determined according to the TNM classification system of the International Union Against Cancer (UICC), and additionally characterized using the Japan Pancreas Society classification (Table I). Thirty-two patients did not receive postoperative chemotherapy, and 31 patients received adjuvant chemotherapy after surgery. Thirteen patients received Uracil/Tegafur (UFT) and 18 patients received gemcitabine (GEM). The median follow-up period was 14.7 months. This study was conducted in accordance with the principles embodied in the 2008 Declaration of Helsinki, and informed consent for the usage of pancreatic tissues was obtained from each patient. Normal pancreatic tissues were obtained from human digestive tissue sets and Human Tissue Microarray 1.

### Human PDAC cell lines

PANC-1, MIA PaCa-2, KLM-1, PK-1, PK-8, PK-9 and PK-59 PDAC cell lines were obtained from the Cell Resource Center for Biomedical Research, Institute of Development, Aging and Cancer, Tohoku University (Sendai, Japan), and Capan-1 was purchased from American Type Culture Collection (ATCC). The cells were grown in RPMI-1640 medium containing 10% fetal bovine serum (FBS), 200 U/ml penicillin, and 200 μg/ml kanamycin at 37°C under a humidified 5% CO_2_ atmosphere. Capan-1 was grown in the same medium containing 15% FBS.

### Quantitative real-time PCR analysis

Total RNA extraction from tumor cells was performed using the RNeasy Mini kit. cDNA synthesis was performed using the Transcriptor First Strand cDNA Synthesis kit following the manufacturer’s protocol. Quantitative real-time PCR (qRT-PCR) was performed using a LightCycler-FastStart DNA Master SYBR Green I system. The primers used for MMP-9 corresponded to nucleotides 192–214 (5′-CAG-AGA-TGC-GTG-GAG-AGT-CGA-AA-3′) and nucleotides 426–445 (5′-GGC-AAA-GGC-GTC-GTC-AAT-CA-3′) of the human MMP-9 cDNA (254 bp, NM_004994). The primers used for 18S rRNA (RS-18) corresponded to nucleotides 184–207 (5′-AAA-GCA-GAC-ATT-GAC-CTC-ACC-AAG-3′) and nucleotides 319–341 (5′-AGG-ACC-TGG-CTG-TAT-TTT-CCA-TC-3′) of the human RS-18 cDNA (158 bp, NM_022551). PCR reaction mixture contaning 2 μl of template cDNA, 3 mM MgCl_2_, 0.5 μM primers, and LightCycler-FastStart DNA Master SYBR Green I mix was applied to the capillary tube (Roche). qRT-PCR was conducted in a LightCycler (Roche) and the PCR products were analyzed using LightCycler Data Analysis software version 3.5 (Roche). The optimized program involved denaturation at 95°C for 10 min, followed by 45 cycles of amplification: 95°C for 10 sec, 60°C for 10 sec, and 72°C for 10 sec for MMP-9; and 95°C for 10 sec, 65°C for 10 sec, and 72°C for 7 sec for RS-18. To confirm amplification specificity, PCR products were subjected to a melting curve analysis. Results were expressed as MMP-9/RS-18, as an internal standard concentration ratio. Each experiment was performed twice, and gene expression measurements were performed in triplicate.

### Immunohistochemistry

Paraffin-embedded tissue sections (3.5 μm) were subjected to immunostaining using the Histofine Simple Stain Max PO (G), (M), or (R) kit. After deparaffinization, endogenous peroxidase activity was blocked by incubation with 0.3% hydrogen peroxide in methanol for 30 min, and the sections were incubated with the appropriate antibody overnight at 4°C (1:50 dilution for the anti-KGF antibody, 1:1000 dilution for the anti-KGFR antibody, and 1:50 dilution for the anti-MMP-9 antibody) using PBS containing 1% BSA. The anti-KGFR antibody used in this study was an affinity-purified rabbit polyclonal antibody raised against a peptide corresponding to an amino acid sequence from the human KGFR protein (32). Bound antibodies were detected with Simple Stain Max PO (G), (M), or (R) reagents using diaminobenzidine tetrahydrochloride (DAB) as the substrate, and the sections were counterstained with Mayer’s hematoxylin. Negative control studies were performed by omitting the primary antibodies. The immunohistochemical results for KGF, KGFR and MMP-9 were evaluated as follows: when staining was noted in the cytoplasm and/or membrane of more than 30% of the tumor cells, regardless of the intensity of staining, the cells were designated positive (32). Two investigators (K.C. and T.I.) separately evaluated all specimens in a blinded manner.

### Effects of rhKGF on MMP-9 mRNA expression

MIA PaCa-2 cells (1×10^5^/well) plated in 6-well plates were grown in 2 ml of RPMI-1640 medium with 10% FBS for 24 h, and then cultured in serum-free medium for 48 h. Subsequently, the cells were cultured in serum-free RPMI-1640 medium in the absence or presence of 10 ng/ml rhKGF for 3 h.

### Transient transfection of short hairpin RNA (shRNA) for KGF

The shRNA for KGF (KGF shRNA) and negative control shRNA were obtained as previously described (32). Twenty-four hours before transfection, KLM-1 cells (2×10^5^/well) were seeded in 6-well plates and grown in 2 ml of RPMI-1640 medium with 10% FBS. Transfection of KGF shRNA and control shRNA were performed using FuGENE HD transfection reagent, according to the manufacturer’s instructions. The medium from KLM-1 cells was replaced with a serum-free medium 48 h after transfection, and cells were incubated for an additional 48 h. The mRNA levels for KGF and MMP-9 in KLM-1 cells were measured by qRT-PCR. All shRNA experiments were conducted twice, with triplicate determinations for each experiment.

### Cell migration and invasion assay

The cell migration assay was performed in 24-well plates using Transwell permeable supports; pore size was 8.0 μm and pore density was 1×10^5^ pores per cm^2^. Cells (1×10^5^) were resuspended in serum-free medium. The cell suspension (500 μl) was placed in the upper compartment, and the lower compartment was immediately filled with 750 μl of RPMI-1640 medium containing 5% FBS and 0 or 20 ng/ml rhKGF. Migrating cells were fixed in 50% methanol and stained with 0.05% crystal violet. The membranes were mounted on glass slides and manually counted in nine different fields under a light microscope at 400× magnification. Each experiment was performed in triplicate.

Invasion assays were conducted using Matrigel-coated Transwell permeable supports. Subsequently, 1×10^5^ cells were resuspended in serum-free medium. The cell suspension (500 μl) was placed in the upper compartment, and the lower compartment was immediately filled with 750 μl of RPMI-1640 medium containing 5% FBS and 0 or 20 ng/ml rhKGF. After 24 h of incubation, the non-invading cells were removed from the upper surface of the separating membrane by gentle scrubbing with a cotton swab. The incubation, staining, counting, and photography procedures were performed as for the migration assay. Each experiment was performed in triplicate.

### Statistical analysis

Whenever indicated, the χ^2^ test and Fisher’s exact test were used to analyze the correlation between KGFR, KGF, or MMP-9 expression and clinicopathologic features. Cumulative survival rate was calculated using the Kaplan-Meier method, and the significance of differences in survival rate was analyzed using the log-rank test; p<0.05 was considered significant in all analyses. Computations were performed using the StatView J software package, version 5.0 (SAS Institute, Inc., Cary, NC, USA).

## Results

### qRT-PCR analysis of MMP-9 in PDAC cell lines

**Figure 1:**
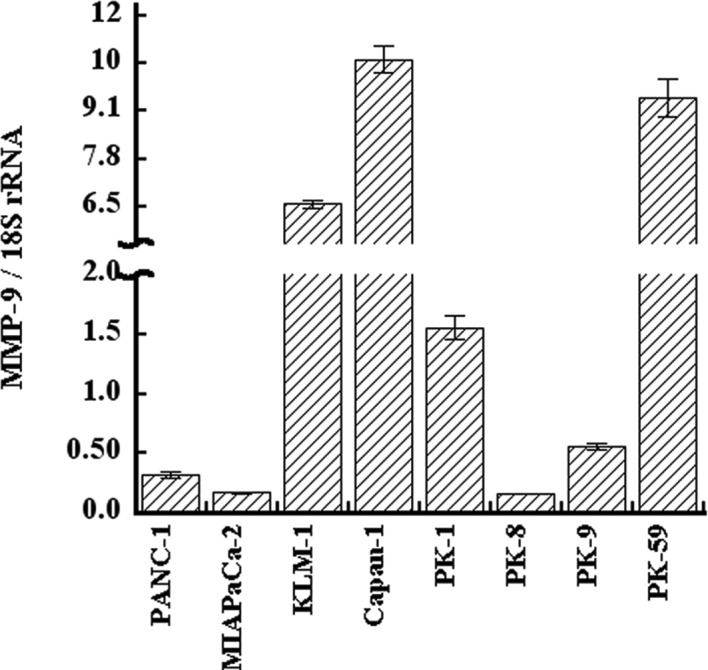
qRT-PCR analysis of MMP-9 in pancreatic cancer cells. Total RNA was extracted from eight pancreatic cancer cell lines and qRT-PCR was performed. MMP-9 mRNA was expressed in all the cancer cell lines at varying levels, and the expression levels had the highest measurements in Capan-1.

Expression of MMP-9 was examined in PANC-1, MIA PaCa-2, KLM-1, Capan-1, PK-1, PK-8, PK-9, and PK-59 cell lines. MMP-9 mRNA was expressed in all eight PDAC cell lines at varying levels. The expression level of MMP-9 mRNA was high in Capan-1, PK-59, and KLM-1 cells, and low in PK-8, MIA PaCa-2, and PANC-1 cells (Fig. 1). The expression level of MMP-9 mRNA in Capan-1 was 66-fold higher than that of PK-8. KGF expression was also high in KLM-1 and Capan-1 cells, and low in MIA PaCa-2 and PANC-1 cells, as previously reported (32). Furthermore, KGFR expression was high in KLM-1 cells and low in MIA PaCa-2 and PANC-1 cells.

### Immunohistochemical analysis of KGF, KGFR, and MMP-9 in PDAC tissues

**Figure 2:**
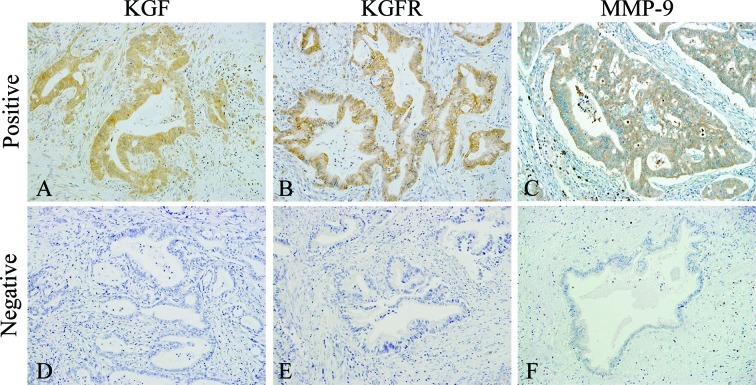
Immunohistochemical analysis of KGF, KGFR and MMP-9 in pancreatic cancer tissues. (A-C) Characteristic staining patterns of KGF, KGFR and MMP-9 in human pancreatic cancer cases. (A) KGF immunoreactivity was detected in the cytoplasm of cancer cells and adjacent stromal fibroblasts. (B) KGFR immunoreactivity was detected in the cytoplasm and cell membranes of cancer cells. (C) MMP-9 immunoreactivity was detected in the cytoplasm of cancer cells. (D-F) KGF-, KGFR- and MMP-9-negative cases. Immunohistochemistry, KGF (A and D), KGFR (B and E) and MMP-9 (C and F); original magnification, ×200.

Immunohistochemical analysis of the PDAC samples was conducted next to determine whether there was a correlation between cellular KGF, KGFR, and MMP-9 expression and clinicopathological features. KGF was localized in the cytoplasm of cancer cells in 27 of 63 (42.9%) patients (Fig. 2A), while KGFR immunoreactivity was detected in the cytoplasm and/or cell membrane of cancer cells in 23 of 63 (36.5%) patients (Fig. 2B). There was a statistically significant correlation between the presence of venous invasion and either KGF (p=0.0065) or KGFR (p=0.050) immunoreactivity (Table I). Moreover, concomitant expression of KGF and KGFR was observed in 14 of 63 (22.2%) samples, and this concomitant expression also correlated with venous invasion (p=0.014, Table I). These results were consistent with our previous report (in a smaller number of cases) that KGF/KGFR expression in human PDAC tissues correlated with venous invasion (32).

We next sought to determine whether KGF/KGFR correlated with MMP-9 expression in human PDAC tissues, because MMP-9 is known to participate in the degradation of type IV collagen, which is a major component of vascular basement membranes. In the normal pancreas, MMP-9 was occasionally detected in the cytoplasm of pancreatic ductal cells, islet cells, and acinar cells, as previously reported (30) (data not shown). In the PDAC samples, MMP-9 immunoreactivity was observed in the cytoplasm of cancer cells in 35 of 63 (55.6%) patients (Fig. 2C). The presence of MMP-9 in cancer cells correlated with venous invasion (p=0.0082, Table I). Moreover, there was a statistically significant correlation between the presence of liver metastasis and either KGF (p=0.00030) or MMP-9 (p=0.022) immunoreactivity (Table II). KGFR expression in PDAC cells did not correlate with liver metastasis.

### Cumulative Kaplan-Meier survival curve and multivariate analysis

**Figure 3:**
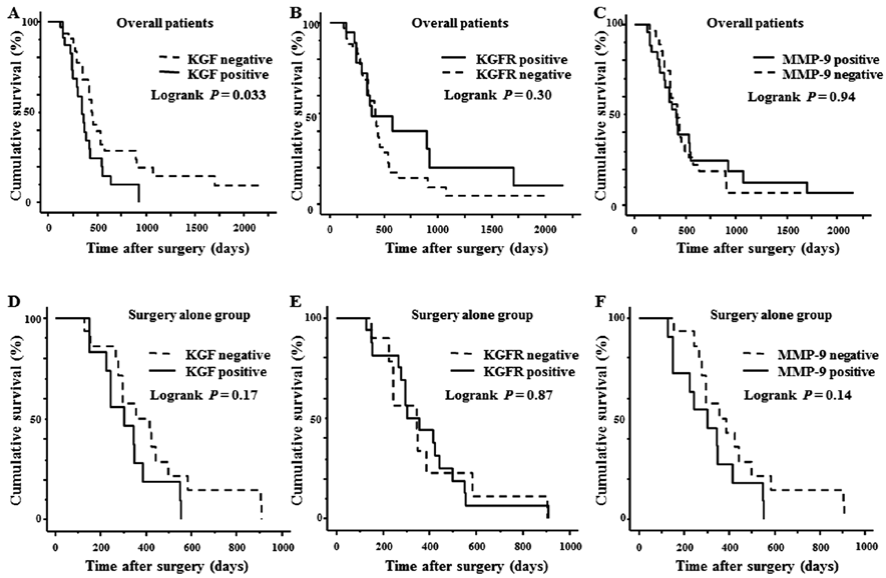
Cumulative Kaplan-Meier survival curves. (A) Curves for all patients with KGF-positive or negative tumors. (B) Curves for all patients with KGFR-positive or negative tumors. (C) Curves for all patients with the MMP-9-positive and negative tumors. (D-F) Curves for the subgroup of patients who only underwent surgical treatment. Statistical significance was only observed in the patients whose tumors were positive for KGF (P=0.033; A).

The overall 2-year survival rate of all 63 PDAC cases was 15.9%. The survival duration of the KGF-positive group was significantly shorter than that of the KGF-negative group (p=0.033; Fig. 3A). The survival rates of the KGFR-positive group and KGFR-negative group were not significantly different (p=0.30; Fig. 3B). Likewise, the survival rates of PDAC patients whose cancer cells were positive for MMP-9 and negative for MMP-9 did not differ significantly (p=0.94; Fig. 3C). Next, the survival rates of patients who had surgery but did not receive any adjuvant chemotherapy were analyzed in a similar manner. In these patients, there was no statistically significant difference between the KGF-, KGFR-, or MMP-9-positive groups and the corresponding negative groups (Fig. 3D-F).

### Effect of KGF on MMP-9 expression in PDAC cells

**Figure 4:**
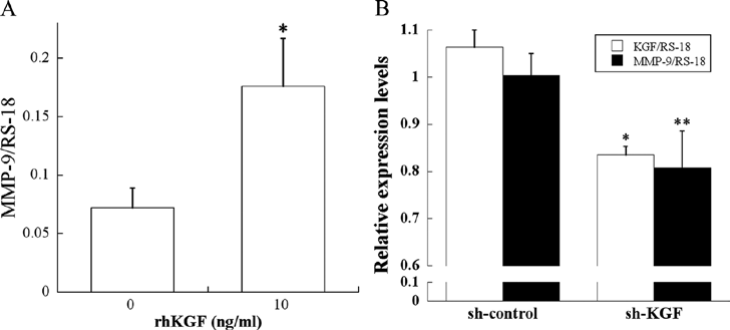
Effect of KGF on MMP-9 expression in pancreatic cancer cells. (A) qRT-PCR analysis showed significant increases in MMP-9 mRNA levels at 3 h after the addition of 10 ng/ml rhKGF to MIA PaCa-2 cells (p<0.05). (B) Effects of sh-KGF on the expression of MMP-9. qRT-PCR revealed that KGF shRNA significantly reduced KGF and MMP-9 mRNA levels in KLM-1 cells compared with the control shRNA (p<0.001 and p<0.05, respectively).

Immuno-staining and survival results pointed to a close relationship between the KGF/KGFR pathway and MMP-9. Therefore, two different types of experiments were performed to assess the relationship between the KGF/KGFR pathway and MMP-9. In the first set of experiments, MIA PaCa-2 cells, which expressed KGFR and negligible levels of KGF, were incubated for 3 h in the absence or presence of rhKGF, and MMP-9 mRNA levels were determined by qRT-PCR. rhKGF caused a significant increase in MMP-9 mRNA levels (p<0.05; Fig. 4A), indicating that exogenous KGF effectively induces MMP-9 expression in PDAC cells.

Next, to establish a better link between KGF and MMP-9 expression, we conducted experiments using KGF shRNA. KGF shRNA and control shRNA were transfected into KLM-1 cells, which expressed the highest KGF levels among all the tested PDAC cell lines (32). qRT-PCR revealed that KGF shRNA significantly reduced KGF and MMP-9 mRNA levels in KLM-1 cells compared with control shRNA (p<0.001 and p<0.05, respectively; Fig. 4B). These results demonstrate that a KGF/KGFR signaling pathway is implicated in MMP-9 regulation.

### Effect of KGF on migration and invasion of human PDAC cells

**Figure 5:**
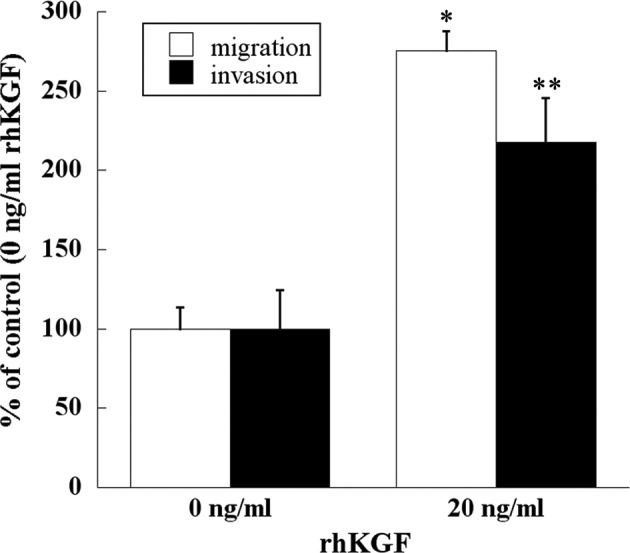
Effects of KGF on migration and invasion of pancreatic cancer cells. The cell migration assay was performed in 24-well plates using Transwell permeable supports and the invasion assays were conducted using Matrigel-coated Transwell permeable supports. Addition of rhKGF significantly enhanced migration and invasion of MIA PaCa-2 cells (p<0.001 and p<0.05, respectively).

Migration and invasion assays were performed to examine whether KGF stimulates the migration and invasion of human PDAC cells. Addition of rhKGF significantly enhanced migration and invasion through the extracellular matrices of MIA PaCa-2 cells (p<0.001 and p<0.05, respectively; Fig. 5).

## Discussion

The strong correlations between MMPs at the protein and mRNA levels and the malignancies of various human cancers have been reported in a number of recent papers (33–37). The presence or elevated expression of MMPs (including MMP-1, -2, -3, -7, -9, -13, and -14) in both primary tumors and/or metastases are positively associated with tumor progression, poor tumor grade, invasive stage of cancer, poor prognosis, metastasis to secondary organs, and shorter survival time (26).

MMP-2 and MMP-9 degrade type IV collagen, and have important roles in the vascular invasion of cancer cells (27). MMP-2 and MMP-9 expression have each been reported in PDAC tissues (38). MMP-9 mRNA was expressed in cancer epithelial cells and stromal cells, while MMP-2 mRNA was predominantly expressed in tumor stromal cells (39). We also examined immunohistochemically the expression of MMP-2 in PDAC tissues. Consistent with previous reports, MMP-2 was mainly localized in stromal tissues adjacent to PDAC cells, but was not localized or weakly localized in PDAC cells. Therefore, we focused on MMP-9 to clarify the relationship between the KGF/KGFR pathway and MMPs in PDAC cells. In the present study, we observed MMP-9 expression at varying levels in PDAC cell lines. KLM-1 cells were established from PK-1 cells as a highly metastatic variant, and the KLM-1 cells metastasize more often than PK-1 cells to the liver of nude mice (40). Notably, MMP-9 expression was higher in KLM-1 cells than in parental PK-1 cells in this study. Furthermore, Capan-1 cells exhibited extremely high MMP-9 levels, and the cells originate from liver metastases in PDAC patients.

In the present study, KGF, KGFR, co-expression of KGF/KGFR, and MMP-9 expression in PDAC tissues are correlated with venous invasion; however, they do not correlate with all other clinicopathological factors. Positive correlation between venous invasion and KGF, KGFR, and co-expression of KGF and KGFR was consistent with our previous study, which contained a smaller number of patients (32). The evidence that expression of KGF and MMP-9 was correlated with liver metastasis may indicate that the KGF/KGFR pathway induces MMP-9 expression, and MMP-9 in turn destroys the basement membrane of tumor vessels to lead to liver metastasis.

To confirm this hypothesis, we examined the effect of KGF on MMP-9 expression using two sets of experiments. First, we exogenously added rhKGF and examined the MMP-9 expression levels in MIA PaCa-2 cells, which express negligible levels of endogenous KGF, but express KGFR. Next, we transfected shRNA-targeting KGF transcripts into KLM-1 cells, which express high levels of KGF and KGFR. Recombinant KGF induced MMP-9 mRNA expression; however, KGF shRNA reduced MMP-9 expression in PDAC cells. These findings suggest that KGF regulates the MMP-9 expression level in PDAC cells. MMPs are considered to be regulated by a variety of cytokines, growth factors, steroid hormones, and phorbol esters (41). However, the transcriptional activation mechanisms underlying this regulation have not been fully understood. Interleukin (IL)-1α, IL-1β, IL-8, TGF β-1, and tumor necrosis factor reportedly induce MMP expression (28). Growth factors implicated in stimulating MMP regulation include basic fibroblast growth factor (bFGF), EGF, and VEGF (42–44). KGF induced MMP-9 expression through NF-kappaB in immortalized/non-tumorigenic human pancreatic ductal epithelial cells (40). To our knowledge, this is a first report that KGF induces MMP-9 expression in PDAC cells. That KGF also stimulates VEGF-A expression in PDAC may suggest that KGF is a strong inducer of tumor vascular angiogenesis and simultaneously stimulates migration and the destruction of incomplete tumor vessels, through the induction of MMP-9 (32,45). In the present study, administration of recombinant KGF to MIA PaCa-2 cells resulted in increased cell migration and invasion through extracellular matrices. These lines of evidence suggest that the KGF/KGFR pathway is centrally involved in vascular invasion and metastases in PDAC.

The relationship between MMP-9 and the prognosis of PDAC patients has been controversial. Some groups have reported no correlation between MMP-9 expression and PDAC prognosis, while others have shown a correlation between MMP-9 expression and poor prognosis (30,31,39,46,47). Furthermore, the expression and roles of KGF/KGFR have not been well clarified. In prostate and bladder cancers, the expression of FGFR2IIIc, another splicing isoform of FGFR2, correlated with more malignant phenotype, compared with KGFR (48,49). Class switching from KGFR to FGFR2IIIc occurs during the metastatic process in prostate cancer. In contrast, KGF/KGFR was closely correlated with venous invasion and liver metastasis in PDAC. Our results indicate that KGF-induced VEGF-A and MMP-9 expression through KGFR might be involved in vascular invasion, liver metastasis, and poor PDAC prognosis. The expression and alteration of other FGFs and/or FGFRs might be involved in discrepancies in the roles of KGF/KGFR in cancer metastasis. Further examination with larger patient populations and additional assessment of other forms of FGFs and FGFRs are needed to clarify the roles of the KGF/KGFR pathway in PDAC metastasis.

In summary, KGF induced MMP-9 expression in KGFR-positive PDAC cells, and KGF and MMP-9 expression were each closely correlated with vascular invasion and liver metastasis of PDAC. The KGF/KGFR pathway may be a novel therapeutic target for PDAC metastasis.

## Figures and Tables

**Table I:** Correlation of clinicopathological features and KGF, KGFR, MMP-9 or co-expression of KGF and KGFR in pancreatic cancers.

		KGF		KGFR		MMP-9		KGF and KGFR	
					
Variables	No.	No. (%)	P-value	No. (%)	P-value	No. (%)	P-value	No. (%)	P-value
Gender									
Male	40	16 (40)	NS	18 (45)	NS	21 (53)	NS	10 (25)	NS
Female	23	11 (48)		5 (22)		14 (61)		4 (17)	
Age									
<65	30	13 (43)	NS	9 (30)	NS	16 (53)	NS	4 (13)	NS
≥65	33	14 (42)		14 (42)		19 (58)		10 (30)	
UICC classification									
T-primary tumor									
T1	4	2 (50)	NS	2 (50)	NS	2 (50)	NS	1 (25)	NS
T2	4	1 (25)		1 (25)		2 (50)		1 (25)	
T3	18	11 (61)		7 (39)		11 (61)		5 (28)	
T4	37	13 (35)		13 (35)		20 (54)		7 (19)	
N-regional lymph nodes									
N0	23	12 (52)	NS	11 (48)	NS	15 (65)	NS	6 (26)	NS
N1	40	15 (38)		12 (30)		20 (50)		8 (20)	
M-distant metastasis									
M0	61	26 (43)	NS	22 (36)	NS	34 (56)	NS	13 (21)	NS
M1	2	1 (50)		1 (50)		1 (50)		1 (50)	
G-histological grading									
G1	34	11 (32)	NS	14 (41)	NS	20 (59)	NS	6 (18)	NS
G2	26	13 (50)		8 (31)		12 (46)		7 (27)	
G3	3	3 (100)		1 (33)		3 (100)		1 (33)	
G4	0	0		0		0		0	
Stage									
I or II	11	6 (55)	NS	5 (45)	NS	5 (45)	NS	4 (36)	NS
III or IV	52	21 (40)		18 (35)		30 (58)		10 (19)	
Other tumor characteristics									
Lymphatic invasion									
Negative	8	4 (50)	NS	3 (38)	NS	5 (63)	NS	2 (25)	NS
Positive	55	23 (42)		20 (36)		30 (55)		12 (22)	
Venous invasion									
Negative	40	12 (30)	0.0065	11 (28)	0.05	19 (48)	0.0082	5 (13)	0.014
Positive	23	15 (65)		12 (52)		16 (70)		9 (39)	
Nerve invasion (intrapancreatic)									
Negative	14	8 (57)	NS	6 (43)	NS	9 (64)	NS	3 (21)	NS
Positive	49	19 (39)		17 (35)		26 (53)		11 (22)	

**Table II:** Correlation of liver metastasis with KGF and MMP-9 expression.

	Liver metastasis		P-value
	Negative (n=32)	Positive (n=28)	
KGF expression			
Negative (n=36)	26	10	0.0003
Positive (n=24)	6	18	
MMP-9 expression			
Negative (n=33)	22	11	0.022
Positive (n=27)	10	17	
